# Supplementary material for: 6-Thioguanine Inhibits Herpes Simplex Virus 1 Infection of Eyes
Source: Microbiol Spectr. 2021 Nov 3;9(3):e00646-21. doi: 10.1128/Spectrum.00646-21 (PMC8567252; doi:10.1128/Spectrum.00646-21)
Supplement: SUPPLEMENTAL FILE 1 — Supplemental material. Download SPECTRUM00646-21_Supp_1_seq13.pdf, PDF file, 0.4 MB [file spectrum00646-21_supp_1_seq13.pdf]

**Supporting Information**

**Fig. S1 Therapeutic concentration of 6-TG is non-toxic in other cell types.**

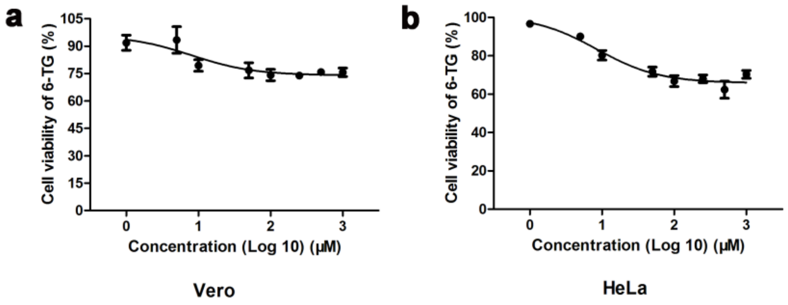


**(a)** Vero or **(b)** HeLa cells were infected with HSV-1 HF in the presence of different concentrations of 6-TG (1, 5, 10, 50, 100, 250, 500 and 1000 μM) for 72 h and their viability was assessed by CCK-8 kit. Data are represented as percentage of untreated cells (n = 4 replicates). One-way ANOVA with Dunnett's multiple comparisons test was performed to determine the significance between 6-TG-treated and untreated cells.

**Fig. S2 The cell viability of a combination of 6-TG and ACV.**

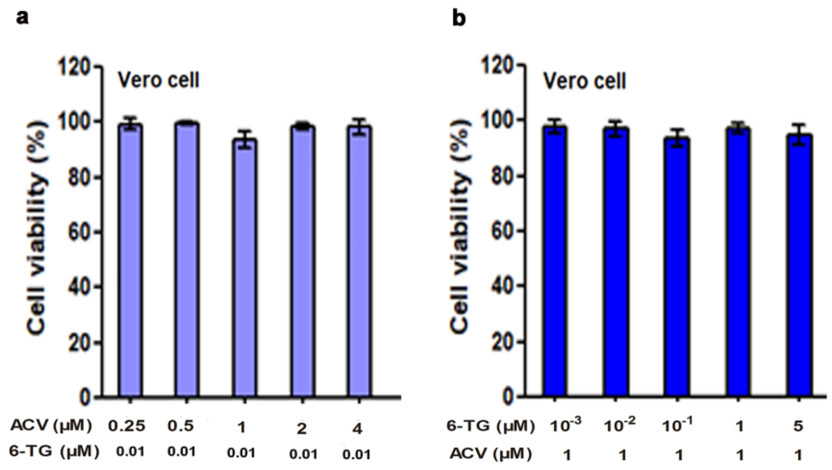


Vero cells were treated with a combination of 6-TG and ACV and cell

viability was assessed by CCK-8 kit (n = 5 replicates). The Vero cells were incubated with the indicated concentrations of 6-TG and ACV together for 24 h and then they were detected by CCK-8 kit and OD value was obtained from a Tecan reader. One-way ANOVA with Dunnett's multiple comparisons test was performed to determine the significance between treated and un-treated cells. Data are presented as mean ± SD from three independent experiments.

**Fig. S3 6-TG did not induce inflammation in the eyes of rabbits.**

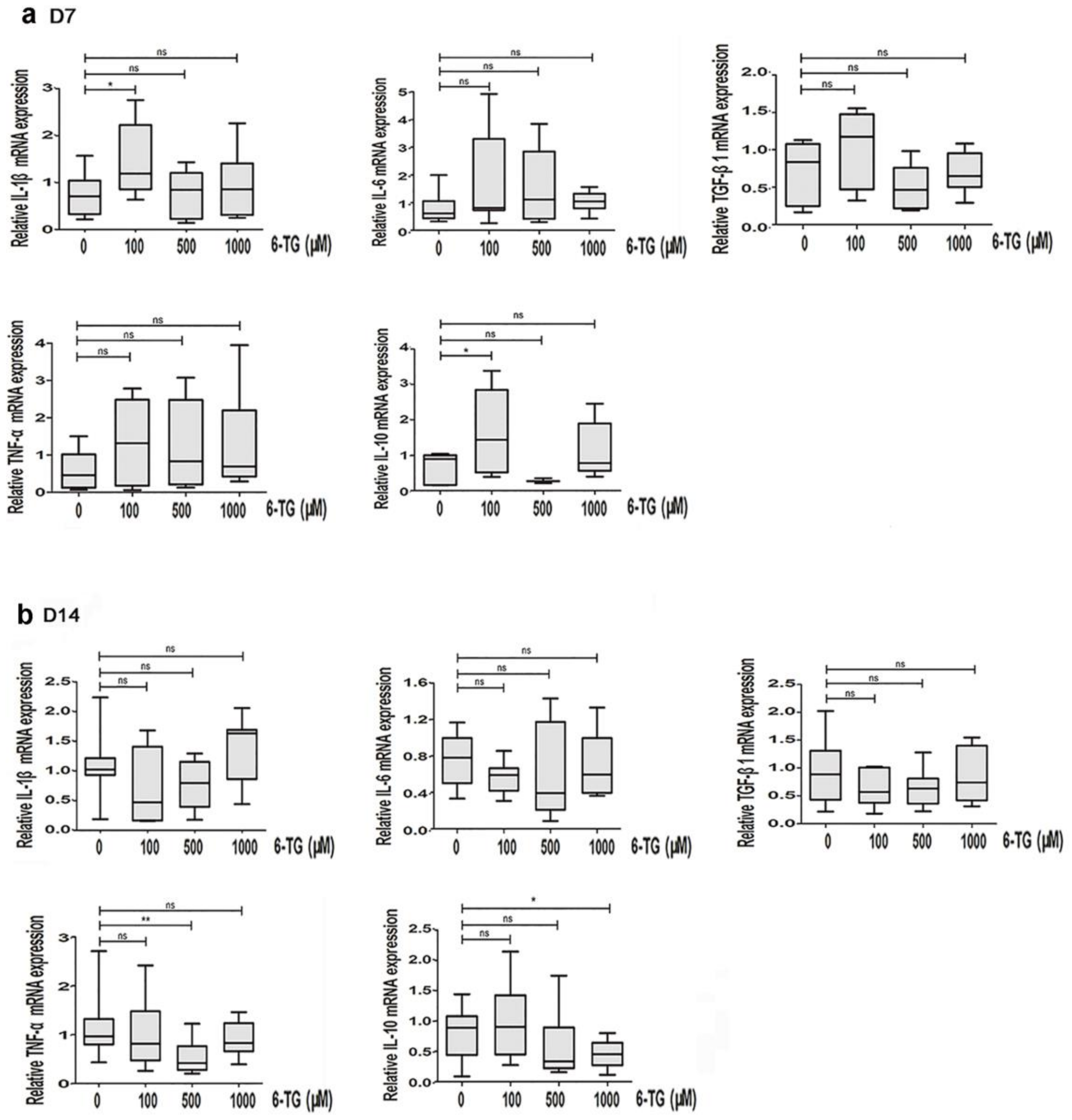


**(a and b)** The corneas were further analyzed of proinflammatory cytokines including IL-1ß, IL-6, TNF-a, IL-10 and TGF-ß on days 7 and 14 by qPCR analysis. The mRNA levels are presented as the relative fold changes compared with those in normal corneas without 6-TG treatment. ns, not significant; All data are representative of three independent experiments.

**Fig. S4 6-TG reduced HSK pathologies in mice.**

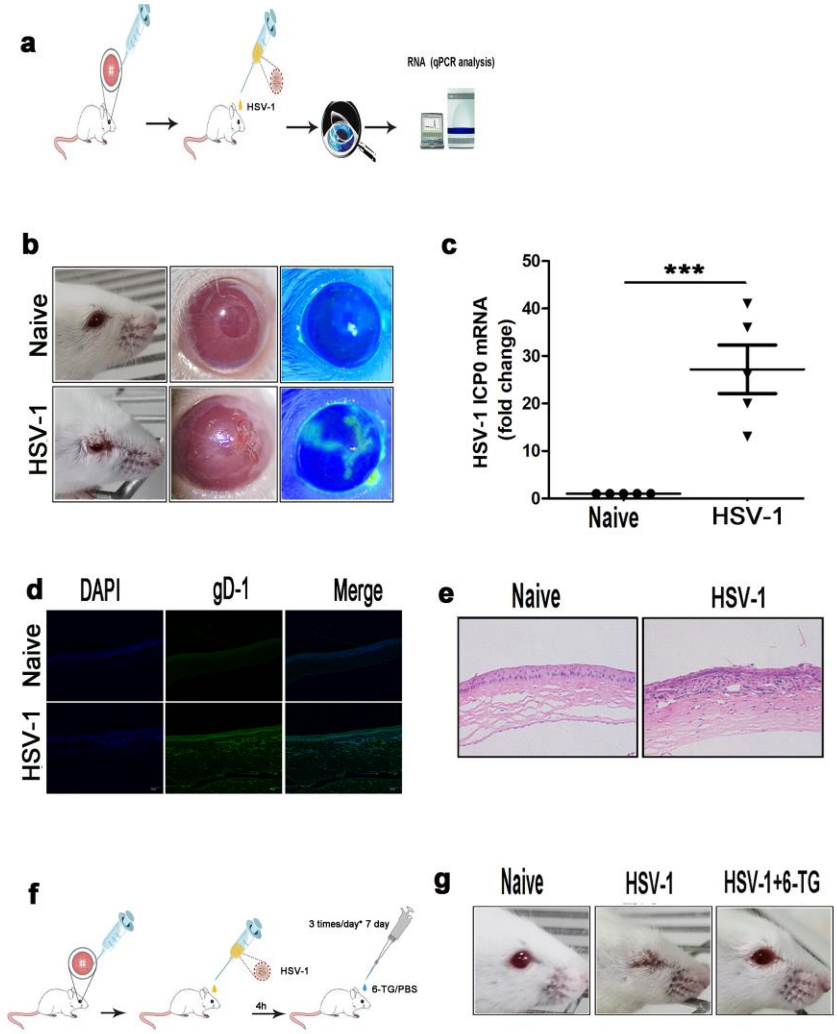


**(a)** The schema picture illustrated the protocol of HSV-1 infected the eyes of the mice. Corneal tissues were collected and analyzed by qPCR analysis. **(b)** Representative photographs were taken from the eyes of mice in the left picture; slit lamp photographs of the mice from HSK group showed edema, opacity or neovascularization of the cornea compared to naïve group in the middle picture; Sodium fluorescein staining showed corneal epithelial damage in the corneal tissues from HSK group in the right picture. **(c)** qPCR analysis of ICP0 mRNA in the

corneal tissues from naïve mice and HSK mice. **(d)** Immunofluorescence staining for gD-1 protein (green) of HSV-1 and DAPI (blue) in the corneal tissues from naïve mice and HSK mice. Magnification (60x). **(e)** Representative corneal histology sections were taken from the eyes of mice at 7 day and corneal thickness was assessed by histology (n = 5). Scale bars:1 mm. **(f)** Mice were inoculated with HSV-1F strain for 4 h in the eyes of the mice and then dropped with 6-TG (200 mg/kg/day) for 7 days. The schema picture illustrated the protocol of HSV-1 infected mice in the presence of 6-TG treatment. **(g)** Representative micrographs of eyes from 6-TG treated HSK mice and PBS-treated-HSK mice.
